# Gold nanoparticle mediated radiation response among key cell components of the tumour microenvironment for the advancement of cancer nanotechnology

**DOI:** 10.1038/s41598-020-68994-0

**Published:** 2020-07-21

**Authors:** Kyle Bromma, Leah Cicon, Wayne Beckham, Devika B. Chithrani

**Affiliations:** 1 Department of Physics and Astronomy, University of Victoria, Victoria, BC Canada; 2 British Columbia Cancer, Victoria, BC Canada; 3 Centre for Advanced Materials and Related Technologies (CAMTEC), University of Victoria, Victoria, BC Canada; 4 Centre for Biomedical Research, University of Victoria, Victoria, BC Canada

**Keywords:** Nanoparticles, Nanotechnology in cancer, Radiotherapy, Targeted therapies, Preclinical research, Double-strand DNA breaks, Endocytosis

## Abstract

One of the major issues in cancer radiotherapy (RT) is normal tissue toxicity. Introduction of radiosensitizers like gold nanoparticles (GNPs) into cancer cells to enhance the local RT dose has been tested successfully. However, it is not known how GNPs interact with other stromal cells such as normal fibroblasts (FBs) and cancer associated fibroblasts (CAFs) within the tumour microenvironment. It is known that FBs turn into CAFs to promote tumour growth. Hence, we used FBs and CAFs along with HeLa (our cancer cell line) to evaluate the differences in GNP uptake and resulting radiation induced damage to elucidate the GNP-mediated therapeutic effect in RT. The CAFs had the largest uptake of the GNPs per cell, with on average 265% relative to HeLa while FBs had only 7.55% the uptake of HeLa and 2.87% the uptake of CAFs. This translated to increases in 53BP1-related DNA damage foci in CAFs (13.5%) and HeLa (9.8%) compared to FBs (8.8%) with RT treatment. This difference in DNA damage due to selective targeting of cancer associated cells over normal cells may allow GNPs to be an effective tool in future cancer RT to battle normal tissue toxicity while improving local RT dose to the tumour.

## Introduction

Cancer is a family of diseases arising from dysregulation of the expression of multiple genes, leading to abnormal cell proliferation and cell death. As a result, there is significant morbidity in patients if left untreated^1^. Before the age of 75, about 1 in 6 people will develop cancer while 1 in 9 will die from it^2^. Aside from surgery, one of the main modalities employed in the treatment of cancer is radiotherapy (RT). RT aims to deliver high doses of ionizing radiation to cancerous tissue, inducing death from damage to important structures such as the DNA or mitochondria^3^. Despite being used in 50% of all patients diagnosed with cancer, one of the major issues in current RT modalities is the normal tissue toxicity in radiosensitive tissue localized closely with the cancer^4,5^. Furthermore, there are many radiobiological hurdles to overcome, such as the influence of cancer stem cells, tumour heterogeneity, tumour hypoxia, metabolic pathways, and other complications, that will increase the radioresistance of the tumour cells^6–8^. While the introduction of targeting methods such as volumetric modulated arc therapy (VMAT) and image guided radiotherapy (IGRT) has improved the efficacy of RT, there is a limit of improvement when it comes to the use of RT as a singular treatment modality^9^. In an effort towards reducing the normal tissue toxicity while increasing the damage to the tumour, radiosensitizers have been introduced^10^.

Radiosensitizers work via various pathways, such as targeting of the radioresistant hypoxic cells in tumours, or through production of reaction oxygen species (ROS)^10,11^. The introduction of high atomic number materials into tumour tissue has been explored as a promising approach to enhance the local radiation dose^12–17^. Among other approaches, use of gold nanoparticles (GNPs) as a radiosensitizer has gained much interest due to their biocompatibility and the feasibility of modulation of size and surface properties for tumour targeting using the enhanced permeability and retention (EPR) effects^17–21^. GNPs have also been shown to be an effective radiosensitizer due to their large cross-section for high energy photon and electron absorption^16,22,23^. Much of the GNP-mediated radiation dose enhancement research has so far focused on cancer cells. However, the role of the tumour microenvironment (TME) has been recently been recognized as a major contributor to tumour propagation^24^. The TME is a complex amalgamation of the extracellular matrix (ECM), stromal cells such as fibroblasts, immune cells, capillaries, and the basement membrane^25^.

**Figure 1:**
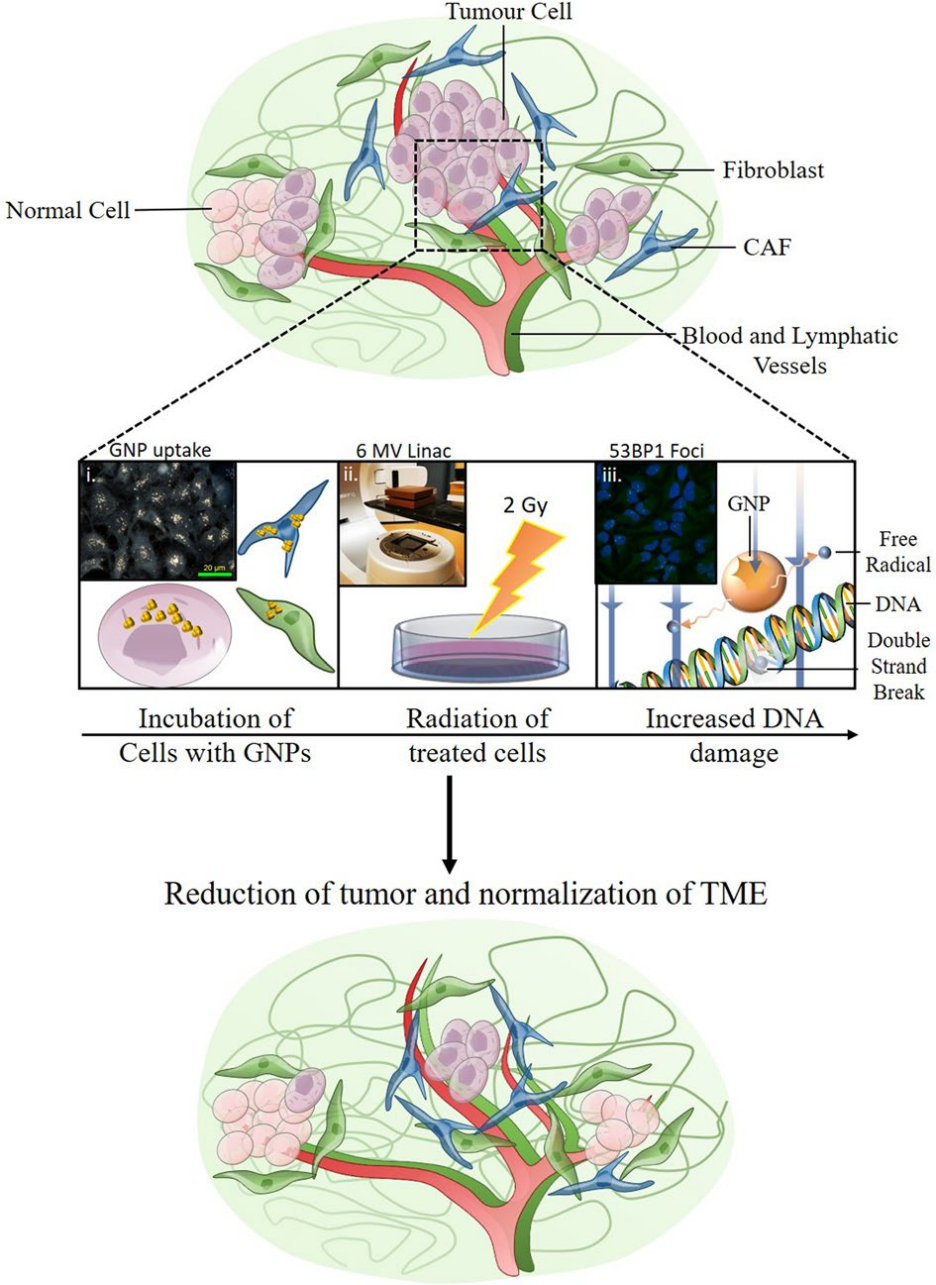
Normal and cancer associated fibroblasts in cancer therapeutics. (**A**) In normal tissue, FBs have anti-tumourigenic properties that supress proliferation of the tumour. However, when CAFs are introduced, a largely pro-tumourigenic influence is now exerted on the tumour microenvironment, leading to growth and eventual metastasis. (**B**) (i) Cells uptake GNPs at differing rates, which is directly related to the efficacy of the treatment. (ii) Cells are irradiated with a 2 Gy dose using a 6 MV linac. (iii) GNPs have been shown to have a radiosensitization effect on cancer cells, through improved formation of free radicals. (**C**) Upon treatment with the dual combination of GNPs and radiation, the TME is more normalized and there is a reduction in tumour growth as well as invasive and migratory behaviour.

Of particular importance to the growth and progression of cancer are fibroblasts^26^. Fibroblasts, specifically activated fibroblasts or myofibroblasts, are associated with the wound healing response^27^. However, these activated fibroblasts can be recruited by the tumour cells, becoming cancer associated fibroblasts (CAFs)^28^. While normal fibroblasts (FBs) have anti-tumourigenic properties, CAFs promote the proliferation of tumours through release of cytokines and chemokines like vascular endothelial growth factors (VEGF) and through ECM remodelling involving matrix metalloproteinases (MMPs)^29,30^. CAFs have also been shown to be related to metastasis and chemoresistance^31,32^. The function of CAFs supports the idea that tumours are ‘wounds that do not heal’ and targeting of CAFs may prove beneficial towards better therapeutic outcomes^33^. Moreover, CAFs have shown to not only improve the radioresistance of the tumour cells but to be highly radioresistant themselves^34^. FBs make up the majority of the tumor stroma (30–60%), depending on the tumor; however, the proportion of differentiated CAFs that make up the stroma is not well known^35,36^. While the tumor-stroma ratio depends on the patient and tumor, when a majority of the tumor is stromal, there is a worse prognosis for a patient^37^. Therefore, focusing only on cancer cells while ignoring the CAFs in TME when optimizing RT-based treatment using GNPs would ignore a large part of what allows tumours to grow and propagate. It is not yet known the cross section of GNP uptake and resulting radiation sensitizing effects across tumour cells, CAFs, and FBs. Hence, this study aims to investigate the GNP-mediated radiation sensitization in three main interrelated cell components in TME as illustrated in the thematic Fig. 1A.

In order to better understand GNP-mediated radiation dose enhancement, we first determined the uptake of GNPs across cancer cells, CAFs, and FBs (Fig. 1B, i). Followed by uptake studies, we proceeded to radiation treatment studies with a clinically relevant 6 MV photon beam as illustrated in Fig. 1B, ii. Our final goal was to map the cell damage due to GNP-mediated radiation dose enhancement by probing DNA double stand breaks (DSBs) (see Fig. 1B, iii). To the best of the author’s knowledge, comparison of GNP uptake and GNP-mediated RT across cancer cells, FBs, and CAFs with GNPs using clinically relevant 6 MV photon beams have not been investigated yet. The use of GNPs to enhance the local dose in the CAFs as well as the tumour could prove to have a significantly beneficial therapeutic outcome. Reducing the ability of CAFs to influence the progression of the tumour such as in Fig. 1C could reduce future complications in patients and improve current treatment modalities. As discussed, normal FBs are recruited into CAFs to promote tumour progression. This study will also elucidate the extent of NP uptake and resulting radiosensitization effects in normal cells compared to other cancer associated cells within TME. Thus, our study will emphasize the importance of considering key cell components of the TME when integrating GNPs into current RT protocols for improved outcome in cancer patients in the near future.

## Results

### Characterization of gold nanoparticle complex (${\text{GNP}}_{{{\text{PEG}} - {\text{RGD}}}} )$

**Figure 2:**
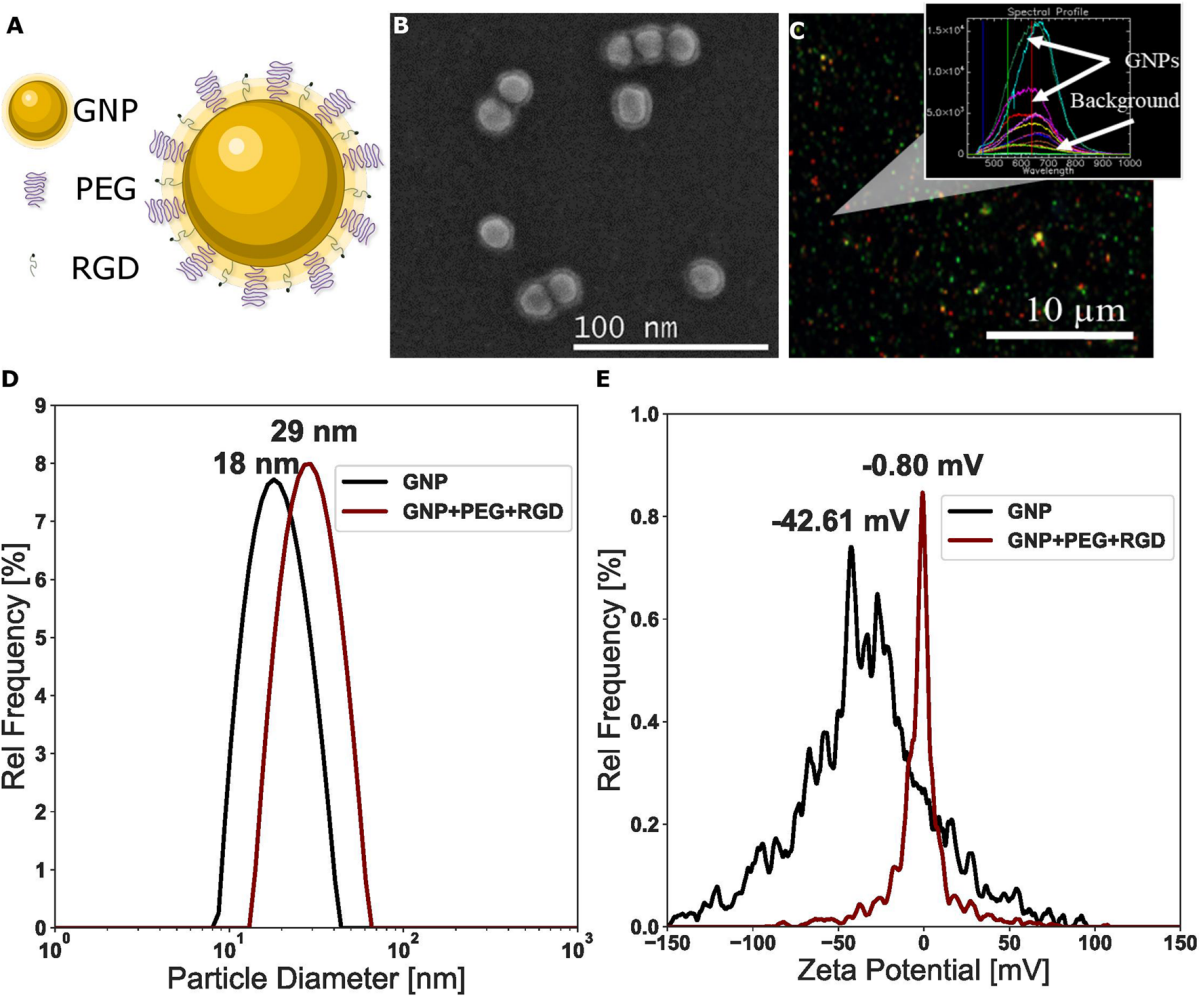
Characterization of gold nanoparticles (**A**) Schematic diagram of the GNP and all the ligands used to form the ${\text{GNP}}_{{{\text{PEG}} - {\text{RGD}}}}$ complex. (**B**) Secondary electron TEM images of ${\text{GNP}}_{{{\text{PEG}} - {\text{RGD}}}}$ complex. (**C**) Darkfield image of GNPs overlayed with spectrum measured using hyper spectral imaging. The GNPs have a clear spectrum relative to background. (**D**) Hydrodynamic diameter from DLS and (**E**) ζ-potential of the GNPs before and after conjugation with PEG and RGD.

The GNPs used for this study were conjugated with polyethylene glycol (PEG) and a peptide containing integrin binding domain RGD (Fig. 2A). The functionalized GNP complex is referred to as ${\text{GNP}}_{{{\text{PEG}} - {\text{RGD}}}} .{ }$ The addition of the PEG molecules prior to RGD peptide is intended as a method to improve stability in the presence of serum, such as media. The use of PEG has been widely documented, and its concentration used in this study is in agreement with literature^38,39^. The GNP formulation was tested, for 24 h, in colorless tissue culture media, as this was the time period the GNPs were in cell culture medium. No significant changes, such as aggregation, to the formulation were observed. Conjugation of the GNPs with PEG and RGD have previously been shown to have improved uptake of PEGylated GNPs^39^. Transmission electron microscopy (TEM) images of the ${\text{GNP}}_{{{\text{PEG}} - {\text{RGD}}}}$ complexes are displayed in Fig. 2B. The average core of the NPs was measured to be a diameter of $17.73\,{\text{nm}} \pm 0.17\,{\text{nm}}$, while the total diameter including surfactants was $20.90\,{\text{nm}} \pm 0.14\,{\text{nm}}.$ Darkfield imaging and the spectrum of each pixel gathered from hyper spectral imaging (HSI) can be seen in Fig. 2C. The spectrum confirms the presence of GNPs and is used to further verify GNP uptake into cells in further experiments. The size, shape, and concentration of the GNPs and GNP complex used in this study were measured using UV–VIS spectroscopy, dynamic light scattering (DLS), and ζ-potential measurements as summarized in Supplement S1A. UV–VIS spectrometry was used to estimate the size and concentration of the GNPs relative to ${\text{GNP}}_{{{\text{PEG}}}}$ and ${\text{GNP}}_{{{\text{PEG}} + {\text{RGD}}}}$ complexes (Supplement S1). UV–VIS has previously been found to be an accurate measurement of the concentration^40^. Further, the efficacy of UV–VIS for measurement of GNP concentration was independently verified through the use of inductively coupled plasma mass spectrometry (ICP-MS), which found that a concentration of 0.2 nM from UV–VIS led to a measured concentration of 0.204 nM. The ratio of the absorbance at the surface plasmon resonance peak to the 450 nm absorbance gave an approximate size of 14–16 nm for both the bare and functionalized GNPs^41^. A slight red shift in the peaks occurred, but the general shape of the spectrum did not change appreciably, signifying stability of the GNP complex.

Further, DLS and ζ-potential were measured before and after the conjugation with PEG and RGD peptide, to verify conjugation (Fig. 2E,F). DLS measurements were completed as well after conjugation with Cy5-thiol-PEG (Supplement S1F), to confirm stability. DLS confirmed the hydrodynamic diameter of the bare GNPs to be 18.02 nm with a polydispersity index of 14.84%, while the ${\text{GNP}}_{{{\text{PEG}} - {\text{RGD}}}}$ complex had a diameter of 29.3 nm and a polydispersity index of 11.08%. The Cy5-thiol-PEG ${\text{GNP}}_{{{\text{PEG}} - {\text{RGD}}}}$ complex had a hydrodynamic diameter of 37.01 nm with a polydispersity index of 15.68%. This increase in the hydrodynamic radius is consistent with conjugation of the different moieties. Further, the difference in the size of the fluorescent GNPs is most likely due to the larger PEG moiety (3.4 kDa for Cy5 vs. 2 kDa for normal). The ζ-potential of the bare GNPs and ${\text{GNP}}_{{{\text{PEG}} - {\text{RGD}}}}$ complex was measured to be $- 42.61\,{\text{mV}} \pm 1.98\,{\text{mV}}$ and $- 0.80\,{\text{mV}} \pm 0.39\,{\text{mV}}$, respectively. This is due to the replacement of the negatively charged citrate molecules with neutral PEG molecules and the positively charged RGD peptides. The ${\text{GNP}}_{{{\text{PEG}} - {\text{RGD}}}}$ complex was also measured for stability in phosphate buffered saline (PBS) at a concentration of 0.2 nM, as seen in Supplement S1E. The GNPs were stable in PBS, with a similar hydrodynamic diameter of 29.42 nm and a polydispersity of 14.54%. Previous studies have shown that GNPs tagged with ~ 1 PEG/nm^2^ surface area demonstrated the best stability, which is the capping density employed in this study^38^.

### Cellular uptake of (${\text{GNP}}_{{{\text{PEG}} - {\text{RGD}}}} ){ }$ complex

**Figure 3:**
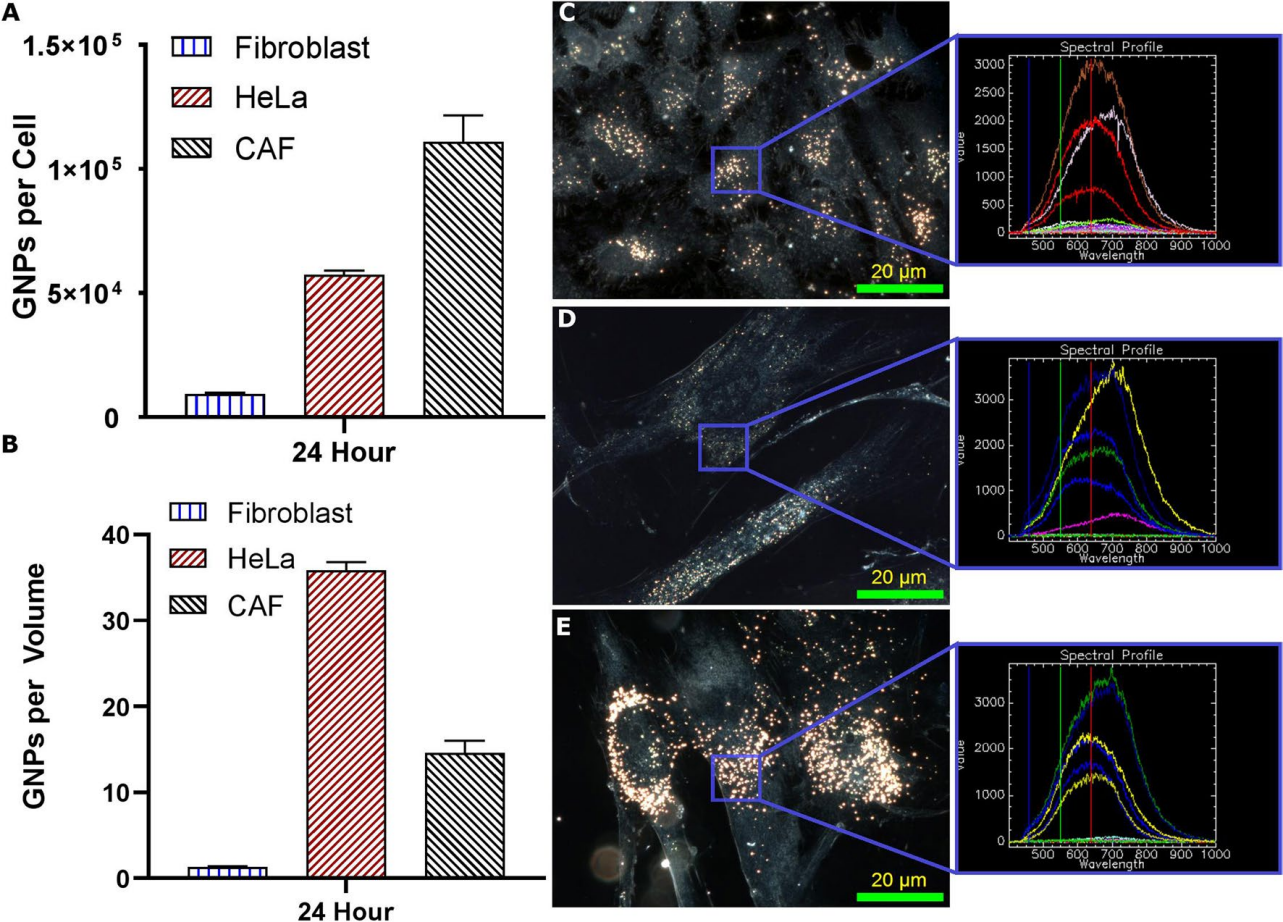
Uptake and retention of gold nanoparticles. (**A**, **B**) Quantification of uptake of 0.2 nM GNPs into all three cell lines as measured using ICP-MS. Error bars are standard deviations from triplicate measurements. (**A**) is GNP per cell, while (**B**) is normalized to the average volume of each cell line. (**C**–**E**) Darkfield images of the GNPs encapsulated in the three cell lines with HeLa on top, FBs in the middle, and CAFs on the bottom. Overlay: the spectrum from HSI is matched in each sample to that of GNPs previously measured. Images taken after 24 h of GNP exposure. Experiments were repeated three times and the data presented is the average. The error bars represent standard deviation. Scale bar = 20 µm.

We chose HeLa as our model cancer cell line while CAFs and FBs were selected as our other two main types of cells in the TME (see Fig. 1). HeLa was chosen as our model cancer cell line as it is highly characterized and simple to model. In order to map the GNP uptake cross section among these three cell lines, we incubated them with ${\text{GNP}}_{{{\text{PEG}} - {\text{RGD}}}}$ complex at a concentration of 0.2 nM for 24 h. The uptake of the ${\text{GNP}}_{{{\text{PEG}} - {\text{RGD}}}}$ complex was quantified using ICP-MS (Fig. 3A). The CAFs had the largest uptake of the GNPs per cell, with on average 265% as many GNPs relative to HeLa, which had the second largest GNPs/cell. FBs had, on average, only 7.55% the uptake of HeLa and 2.87% the uptake of CAFs. We also presented the GNP update data per unit volume of the cell (Fig. 3B). The average volume per unit of CAFs, FBs, and Hela were $8181\,\upmu {\text{m}}^{3} \pm 82\,\upmu {\text{m}}^{3}, 7700\,\upmu {\text{m}}^{3} \pm 77\,\upmu {\text{m}}^{3},\,{\text{and}}\, 2205\,\upmu {\text{m}}^{3} \pm 22\,\upmu {\text{m}}^{3}$, respectively. We found that CAFs have 50% of the average GNPs/volume found in HeLa, while FBs have 5% of the GNPs found in HeLa (Fig. 3B). Similar results are seen when adjusting for GNPs per surface area (Supplement S2A). We have also looked at the ability of these cells to retain the internalized GNPs once the tissue culture media was replaced with fresh media (no GNPs). After 24 h of incubation with the fresh media, it was found that retention of the ${\text{GNP}}_{{{\text{PEG}} - {\text{RGD}}}}$ complexes was higher in cancer cells and CAFs (~ 65%) when compared to FBs (~ 45%) (Supplement S2B).

**Figure 4:**
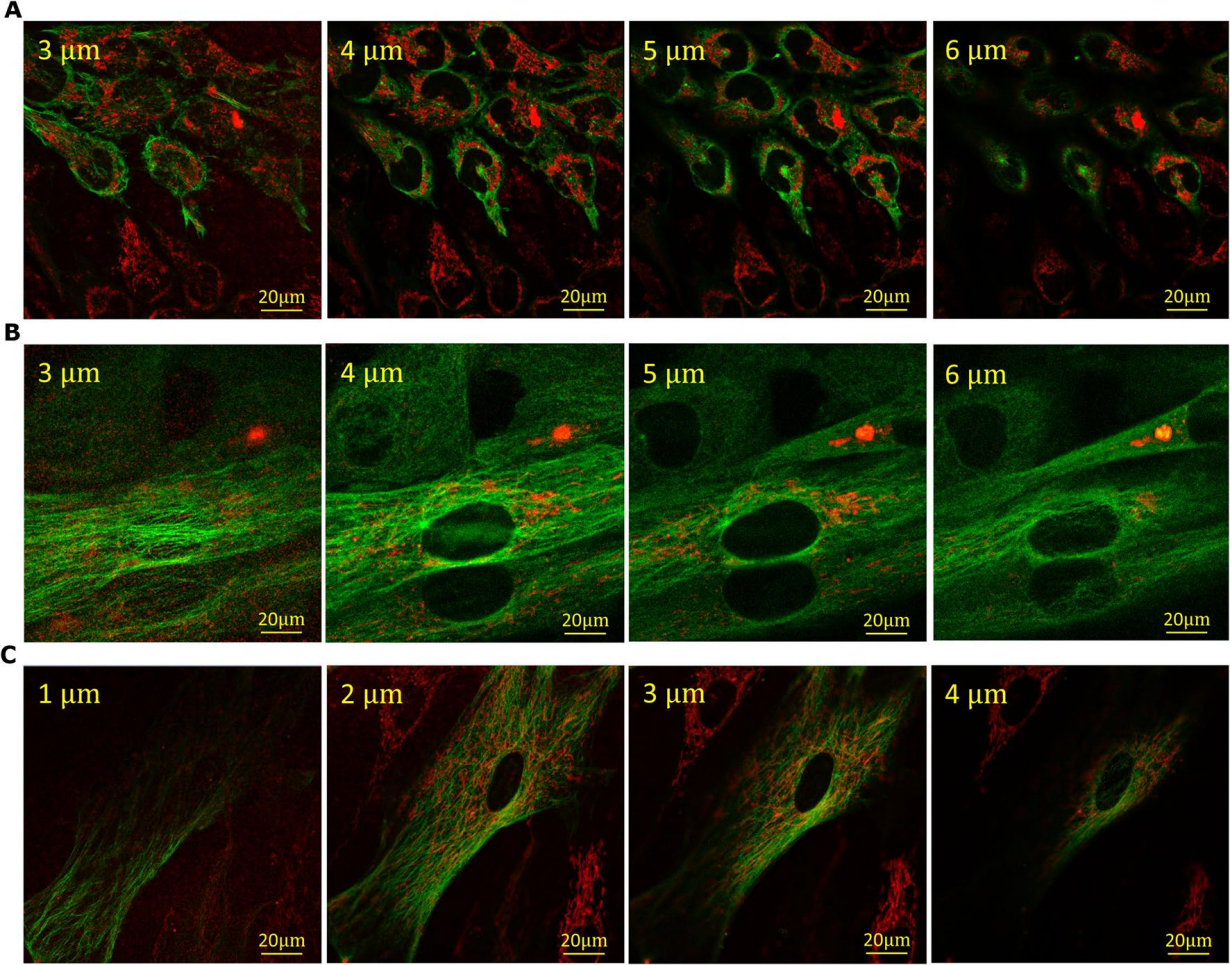
Z-stack using confocal imaging of three cell lines. (**A**–**C**) Confocal images of distribution of GNPs (marked in red) and microtubules (marked in green) in Hela, FBs, and CAFs throughout a z-stack of the cells 24 h after treatment. (**A**) HeLa cells proliferate quickly, thus many of the cells do not have the microtubule viral stain. GNPs tend to be bundled close to the nuclei. (**B**) FBs are much larger cells compared to HeLa and have relatively low GNP content. (**C**) CAFs are comparatively similar in size to FBs, but qualitatively have much larger uptake, gathered around the nuclei and throughout the cell. Scale bar = 20 µm. Top left corner is depth in cell for each slice.

GNP uptake was imaged using darkfield microscopy in combination with HSI as seen in Fig. 3C–E. The combination of darkfield microscopy with HSI imaging allows for a more complete analysis of a sample with GNPs, including mapping of GNPs and verification of the materials prescence^42^. Qualitatively, the cells appear to follow the same trend as quantitative data in Fig. 3A. Furthermore, the GNPs appear to localize closer to the nuclei in the CAFs and HeLa while being more distributed throughout the cells in the FBs. It is known that the vesicles and organelles in the cells are transported along the microtubules (MTs) within the cells^43^. Similarly, vesicles containing GNPs within the cell have previously been shown to travel along the MTs following uptake^44^. The process by which this occurs is explained in Supplement S3. Hence, we also looked at the MT network and distribution of vesicles containing GNPs across these three cell lines using confocal microscopy (see Fig. 4). The MT structure of the cells was tagged with a viral transfection stain containing the green fluorescent protein (GFP) while the ${\text{GNP}}_{{{\text{PEG}} - {\text{RGD}}}}$ complexes were tagged with the red fluorescent Cy5. These images in Fig. 4 showcase the increased uptake of GNPs in HeLa and CAFs relative to FBs, which agrees with quantitative data in Fig. 3.

### Effect of radiation on cell proliferation

**Figure 5:**
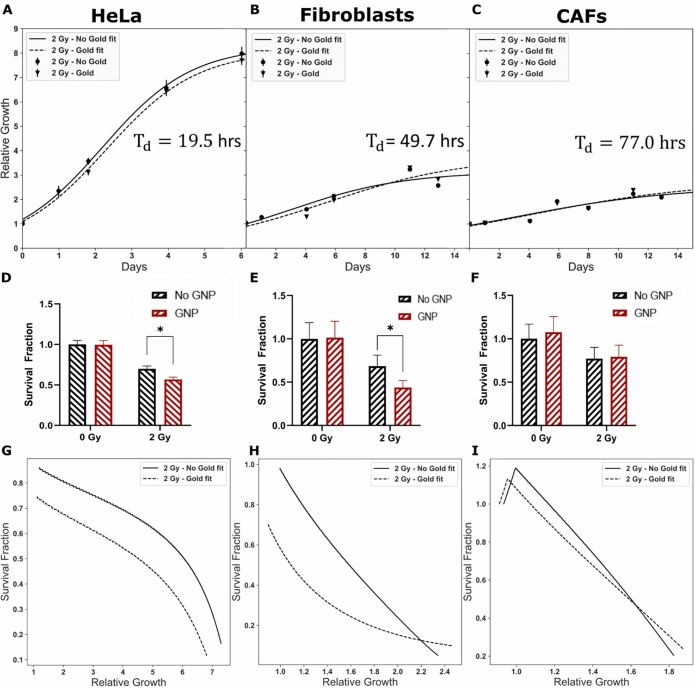
Proliferation assay. (**A**–**C**) Reparametrized logistic curve fit (line) using non-linear least square to collected data (points). (**D**–**F**) Survival fraction estimated from delay in growth curve after one doubling time. * represents a significant difference as explained in “Materials and methods” section. (**G**–**I**) The change in calculated survival fraction as a function of relative growth. A crossover indicates return to control growth. (**A**, **D**) HeLa doubling time is estimated from the control curve (${\text{T}}_{{\text{d}}} = 19.5$ h), where the SF was calculated from a delay time estimated at a relative growth of 2.25. (**B**, **E**) FBs (${\text{T}}_{{\text{d}}} = 49.7\,{\text{h}}$) had a delay time estimated from a relative growth of 1.65, from which the SF was calculated. (**D**, **F**) CAFs (${\text{T}}_{{\text{d}}} = 77.0\, {\text{h}}$) SF was calculated from a delay time estimated from a relative growth of 1.36. Experiments were repeated three times and the data presented is the average. The error bars represent standard deviation.

The gold standard in measuring cell reproductive death following treatment with radiation is the clonogenic assay. The clonogenic assay works by treating cells, letting them grow for the equivalent of 6 doubling times to form colonies. While effective with cell lines such as HeLa that proliferate quickly and in colonies, the FBs and CAFs, proliferate very slowly and do not form clear colonies. Thus, we used cell growth curves to calculate the cell survival fraction. The experimental proliferation or relative growth data for HeLa, FBs, and CAFs were plotted in Fig. 5A-C, respectively. The experimental data was fitted using Eq. (1) to generate parameters for determining the survival fraction (see dotted and solid lines in Fig. 5A–C)^45^. The experimental and fitted data presented in Fig. 5A–C corresponds to cells treated with radiation while corresponding data for non-radiated cells are presented in Supplement S4. The relative growth (y) is described by Eq. (1) where µ is the growth rate, A is the asymptote or maximum growth, and λ is the lag time.

1 $${\text{y}} = \frac{{\text{A}}}{{\left\{ {1 + \exp \left[ {\frac{{4\upmu }}{{\text{A}}}\left( {\uplambda - {\text{t}}} \right) + 2} \right]} \right\}}}$$

The values of fitted parameters such as µ and A from growth curves were used to calculate cell doubling time (T_d_) as outlined in Eq. (2).

2 $${\text{T}}_{{\text{d}}} = \ln \left( 2 \right) \cdot \left( {\frac{{4\upmu }}{{\text{A}}}} \right)^{ - 1}$$

The calculated values of T_d_ for HeLa, FBs, and CAFs were 19.5, 49.7, and 77.0 h, respectively.

Finally, the survival fraction (SF) was calculated using Eq. (3), where T_d_ is the doubling time and ${\text{T}}_{{{\text{delay}}}}$ is the delay time.

3 $${\text{SF}} = 2^{{ - \frac{{{\text{T}}_{{{\text{delay}}}} }}{{{\text{T}}_{{\text{d}}} }}}}$$

${\text{T}}_{{{\text{delay}}}}$ is defined as the time takes to reach the same growth in the treated curves compared to control after one doubling time^46^. The calculated survival fraction corresponding with the relative growth can be seen in Fig. 5D–F. The values corresponding to each cell line’s fit are listed in Supplement S3.

Based on the survival fraction data in Fig. 5D, we noticed a significant decrease in survival (p = 0.0009) of 13.0% when radiated cells are treated with GNPs relative to control for HeLa cells. The cells treated with GNPs do not recover to similar levels as control, as seen in Fig. 5G. At the same time, FBs (Fig. 5E) sees a significant drop in the survival fraction (p = 0.008) of 24.7% after one doubling time when treated with GNPs in the radiated samples, which eventually recovers to similar levels of the non-GNP radiated sample, after > 2 ${\text{T}}_{{\text{d}}}$. CAFs (Fig. 5F), on the other hand, have a significant reduction (p = 0.002) in survival between radiated versus non-radiated samples, but has no significant difference between irradiated samples treated with and without GNPs. The change in calculated survival fraction in radiated CAFs does not change over time (Fig. 5I).

### Probing the radiation damage using DNA double stand breaks (DSB) assay

**Figure 6:**
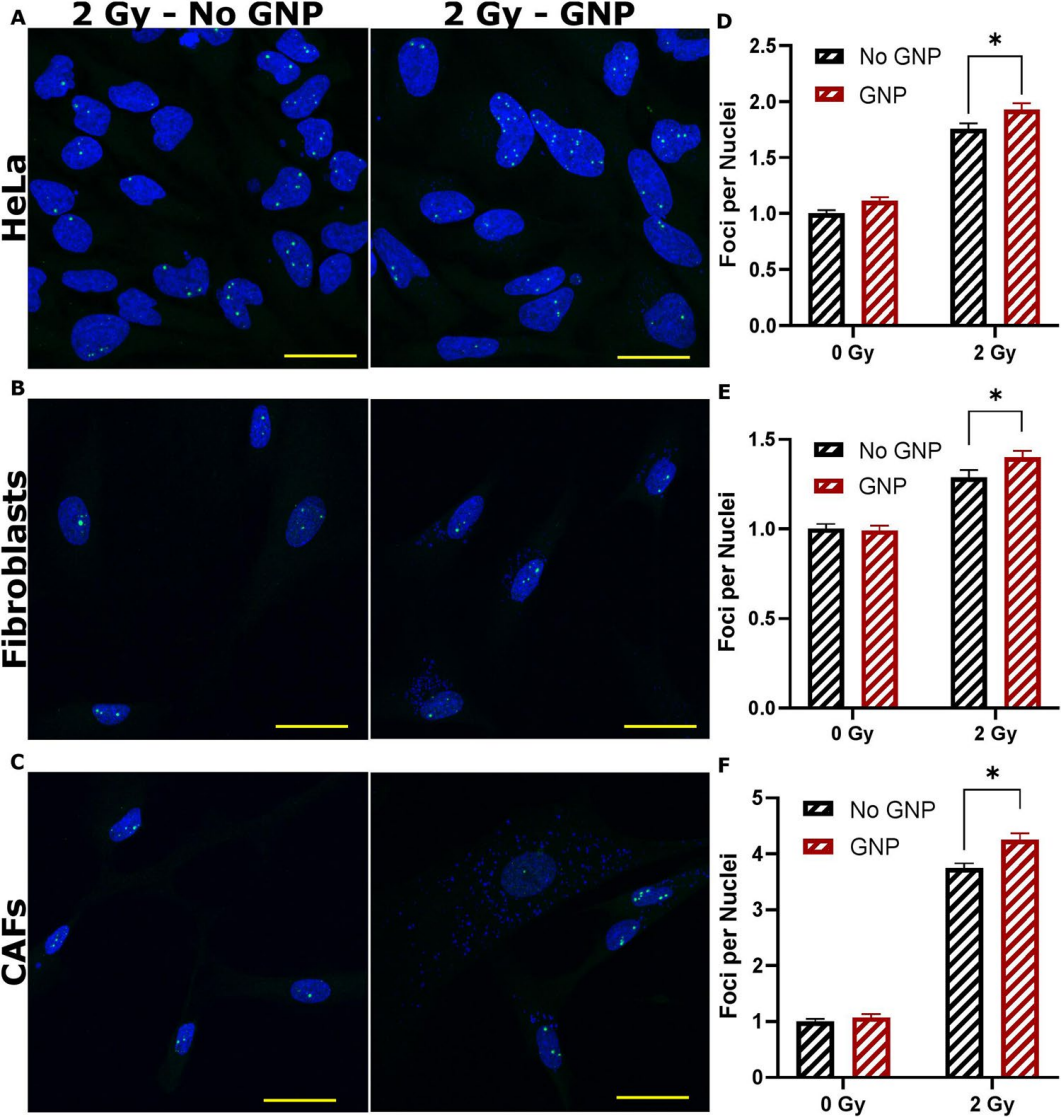
Immunofluorescence assay using 53BP1 foci. (**A**-**C**) Image panels of the three cell lines with 0 Gy and 2 Gy doses, and with or without GNPs. The nuclei are marked in blue while 53BP1 are marked in green. The green dots present in the nuclei are the foci, while the excess staining seen outside the nuclei is a non-specific fluorescence signal. (**D**-**F**) Quantitative data of the number of foci per nuclei, gathered from a minimum 50 cells. * represents a significant difference as described in “Materials and methods” section. Further images are available in Supplement S6A–C. Experiments were repeated three times and the data presented is the average. The error bars represent standard error. Scale bar = 20 µm.

We performed an immunofluorescent assay to further validate the data generated from cell proliferation curves in addition to measuring the survival fraction discussed in the previous section. This was done by probing DNA damage using antibodies against two proteins, 53BP1 and ${\gamma H}2{\text{AX}}$, which are present in the event of a DNA DSB. All three cell lines were treated as described in “Materials and methods” section and imaged using confocal microscopy (Fig. 6A–C). For HeLa (Fig. 6D) and FBs (Fig. 6E), we see a significant increase (*p* = 0.00004; *p* = 0.01) of 9.8% and 8.8%, respectively, in the number of 53BP1 foci formed after irradiating with GNPs vs without GNPs (control), in agreement with previous results. However, for irradiated CAFs, unlike previously measured, we see a significant (*p* = 0.004) increase of 13.5% in the 53BP1 foci formation relative to control, signifying an increase in damage. For all cell lines, there was no significant change in control foci formation when treated with or without GNPs. Results with ${\gamma H}2{\text{AX}}$ can be seen in Supplement S5, with the resulting foci per nuclei in Supplement S6A–C. Furthermore, we verified the presence of the GNPs using pseudo-brightfield imaging from the confocal microscope, which revealed GNP clusters, as seen in Supplement S6.

## Discussion

One of the major limitations in current RT is that the dose cannot be further increased due to normal tissue toxicity. One of the ways to increase the local dose to the tumour is through the introduction of high Z materials such as GNPs^12-21^. Hence, one of the goals of this study was to evaluate the GNP uptake and resulting damage due to radiation in normal cells such as FBs compared to cancer cells. It has been reported that FBs are present within TME and they can turn into CAFs to support tumour growth^28-30^. Hence, if we were to build a successful GNP-based approach to improve current RT, it is necessary to understand the GNP uptake and resulting radiation induced damage across these multiple cell types, not just in cancer cells, as laid out in schematic Fig. 1. The ultimate goal of using GNPs in RT will be to reduce the tumour cells while preserving FBs and minimizing the activity of CAFs.

Size and surface properties of GNPs play a significant role in their cellular uptake, tissue penetration, and residence time. GNPs of size 50 nm have previously been shown to have the best uptake in a monolayer of cells^44^. However, when moving to a more complex environment such as a three-dimensional in vitro structure like spheroids, the use of smaller GNPs of sizes ~ 20 nm have been shown to have improved uptake and penetration when compared with larger NPs, especially those of size 100 nm or larger^47^. Furthermore, the use of GNPs with PEG and RGD of size 15 nm and 50 nm have been tested previously, and, due to the addition of the moieties, GNPs of smaller size had a more optimum uptake, as can be seen in Supplement S2C–D. Thus, towards the goal of optimizing the use of GNPs in a more complex environment, we chose smaller GNPs of diameter 15 nm functionalized with both PEG and RGD peptide^39,48,49^. The GNP complex (${\text{GNP}}_{{{\text{PEG}} - {\text{RGD}}}} )$ was characterized fully as shown in Fig. 2. PEG is an important molecule to be conjugated to the NP surface as it has been shown to improve the stabilization and circulation of macromolecules, facilitating better tumour targeting^50^. However, previous studies have shown that the PEG reduces the uptake of GNPs, and thus a peptide containing integrin binding domain RGD was introduced to counteract this effect; the RGD peptide has been shown to improve endocytosis as well as effectively target tumours^39,49^. HeLa has previously been shown to highly express ${\upalpha v\upbeta }3$, an RGD-specific integrin, and may express at least 2 others, ${\upalpha }5{\upbeta }1$ and ${\upalpha v\upbeta }5$^51^. Further, FB cell lines have been shown to have abundant surface expression of integrins, of a particularly high nature^52^. CAFs have largely been shown to express many different RGD-specific integrins on their surface, partially as a means of crosstalk between cancers cells^53^. However, exact differences in integrin expression between cancer associated cells and FBs used in this study are not fully known yet. Therefore, we will conduct future studies to confirm whether the specific differences in the integrin expression of the cell lines used, if any, could contribute towards the difference in GNP uptake reported in this study.

Our in vitro studies were conducted at a GNP concentration of 0.2 nM or 0.05 $\frac{{{\mu g}}}{{\text{g}}}$, which can be achieved in vivo^17,21,48^. It is also important to note that such concentrations in tumour tissue led to significant GNP-mediated sensitization in vivo^17,21^. Our results show very promising outcomes in the use of GNPs in RT. For example, normal cells (FBs) had the least uptake of GNPs compared to cancer associated cells (HeLa and CAFs). According to Fig. 3C–E, size and shape of these three cell lines were different from each other. We also estimated the volume of each cell type using a Z2 Coulter Counter and Fig. 3B represents number of GNPs per unit volume. The cancer cell line, HeLa, appears to have a denser concentration of GNPs compared to CAFs. This could be due to the overall smaller size of HeLa compared to CAFs. Based on previous research, different cell lines have previously been shown to alter the efficacy of endocytosis of GNPs, explaining some of the measured difference^54^.

Much of the GNP-mediated radiation dose enhancement research has been focused just on cancer cells. Hence, one of the major goals of this study was to assess GNP-mediated radiation damage in other cells within TME such as FBs and CAFs. The clonogenic assay is the gold standard in measuring the radiation induced cell damage^55,56^. The survival fraction generated using clonogenic assay for HeLa is given in Supplement S7. However, it was not possible to generate colonies for measuring survival fraction in the case of FBs and CAFs due to their longer doubling time at 0 Gy (T_d_ (FBs) = 49.7 h and T_d_ (CAF) = 77.0 h). Hence, we used cell proliferation data to generate parameters to calculate the survival fraction, as seen in Fig. 5A–C. Control data for all cell lines and their respective fit parameters can be found in Supplement S4. There was a significant decrease of 13.0% in survival (*p* = 0.0009) corresponding to HeLa cells treated with both GNPs and radiation compared to cells just treated with radiation (control). Most importantly, this decrease in survival was maintained throughout the entire growth curve resulting in a lower survival after few doubling times (see Fig. 5G). This could be very important in fractionated RT which is heavily used in the clinic. This is one of the reasons we chose a fractionated dose of 2 Gy for our experiments. We saw very promising data for FBs (Fig. 5E,H) where there was a significant drop in the survival fraction (*p* = 0.008) of 24.7% due to GNP-mediated RT vs control after one doubling time, which eventually recovered to similar levels of the control irradiated sample, after > 2${\text{T}}_{{\text{d}}}$. This could be important since their survival after the treatment could suppress the progression of the tumour. This highlights the ability of normal tissue to repair, given time, which is the basis for fractionated radiotherapy^57^. Further, the idea of normal tissue recovering is also utilized in chemotherapy, via different dose schedules^58^. In contrast, CAFs (Fig. 5F,I) had a significant reduction (*p* = 0.002) in survival between radiated vs non-radiated samples but has no significant difference between irradiated samples treated with and without GNPs. It has been shown that the proliferation of CAFs is inherently very low. Hence, the use of proliferation data may be not that relevant to measure radiation induced damage. Therefore, we used DNA DSBs assay to probe the radiation induced damage, especially in CAFs.

Mapping of DNA double strand breaks (DSBs) is widely used as another measure of evaluation of the radiation induced damage^19^. We used antibodies against repair proteins such as 53BP1 and ${\gamma H}2{\text{AX}}$ to measure the extent of radiation induced damage 24 h post-radiation^59,60^. By measuring foci formation 24 h after treatment with ionizing radiation, residual damage is captured, representing DNA damage that is unrepaired and likely to lead to loss of clonogenic potential^61^. Furthermore, the number of foci can depend on the type of damage as well as the damage repair mechanism. The 53BP1 foci signal non-homologous end joining (NHEJ) as a repair mechanism, while a lack of the 53BP1 but presence of a ${\gamma H}2{\text{AX}}$ foci could signal homologous repair (HR) in the S/G2 phase^62^. This could lead to a varying number of ${\gamma H}2{\text{AX }}$ foci compared 53BP1 foci. Further to this point, during S phase, there is a pan-nuclear ${\gamma H}2{\text{AX}}$ speckle that can form, leading to miscounting of these foci^62^. Meanwhile, during the S-phase, 53BP1 can still act, via NHEJ, in a H2AX independent manor, suggesting that 53BP1 can function in DNA repair independently of H2AX^63^. High expression of γH2AX can also be suggestive of defective DNA repair pathway and/or genomic instability, whereas 53BP1is a conserved checkpoint protein DNA DSB sensor^64^. For these reasons, solely 53BP1 data is discussed here while ${\gamma H}2{\text{AX}}$ data has been added to Supplement S5 and S6A–C. For all three cell lines studied, there was a statistically significant difference in number of foci per cells after 24 h of radiation for cell treated with GNPs plus RT vs RT alone (Fig. 6). For example, the increase in number of foci for HeLa (Fig. 6D) and CAFs (Fig. 6E) was 9.8% and 13.5%, respectively while it was 8.8% for FBs. The results of the DSB foci assay correlates well with the difference in uptake of GNPs in the different cell lines, with CAF having the largest increase in DSBs while having the most GNPs, followed similarly by HeLa and FBs. The number of GNPs in a cell during radiation is directly correlated to the dose enhancement factor, defined here as the ratio of foci formed in GNP-treated irradiated cells to control irradiated cells^19^. Furthermore, even when correcting for cell volume, where HeLa has a larger ratio of GNPs/volume, the total quantity is arguably more important. As Fig. 3E showcases, the GNPs tend to cluster closer to the nuclei in the CAFs which may improve treatment efficacy: the more GNPs gathered near the nuclei, the larger the resulting dose enhancement upon radiation^43^.

Despite the increase of DNA damage in the irradiated CAFs (Fig. 6), there is no measurable difference or delay in the proliferation of the CAFs treated with GNPs plus RT vs RT alone over time (Fig. 5). This may be a result of CAF cell lines responding to radiation differently that other cell lines^34^. Hellevik et al. found that using stereotactic ablative radiotherapy on CAFs resulted in an increase in 53BP1 foci, but no increase in cell death, even with increasing doses. There was, however, an induced senescence response, as well as compromised migratory and invasive ability with larger doses, correlating with increased 53BP1 foci^65^. Resazurin, the proliferation assay used in this experiment, is directly correlated with cell number^66^. Thus, our proliferation assay may be showing the GNP treated CAFs entering senescence at the same rate as irradiated control, while actual cell death did not occur in either sample. However, despite the lack of cell proliferation, there was a radiosensitization effect present due to the measured increase in DSBs. Increase in DNA DSBs could be due to the enhancement in the absorbed dose due to GNPs^67,68^. Further experiments are required to investigate how the observed increase in DNA DSBs in CAFs affect cancer cell growth in the TME. Understanding of the tumour stroma on GNP-mediated radiosensitization is critical to fully exploit the benefits of this novel therapeutic approach in future cancer treatments. We used a simplified monoculture for this study, in order to better understand important individual components of the TME. In the future, testing of the tumour cells, CAFs, and FBs with GNP-mediated radiotherapy will be done in co-culture. Further, future studies will involve engineering of a three-dimensional models with both cancer cells and CAFs. The next step will then be to extend the study to an in vivo environment to understand the extent of reduction in metastasis and pro-tumourigenesis properties currently present in the TME due to the CAFs.

Targeting of the cancer cells is still the main priority when using GNPs with RT; however, CAFs have been shown to be largely important to the growth and metastasis of the tumor. This study suggests that CAFs have a larger DNA damage response due to the inclusion of GNPs into radiation treatment and thus could prove to be a useful target in the future. An improved tumour response due to selective targeting of cancerous tissue such as CAFs over normal tissue will allow for GNPs to be an effective tool in radiotherapeutics^69^.

## Conclusion

The use of GNPs has been largely explored as a radiation dose enhancer which can effectively increase the dose to the tumour while sparing normal tissue^66^. GNPs have been shown to successfully increase the deposited dose at clinically relevant energies. However, the introduction of the TME with stromal cells such as CAFs adds complexities that cannot be ignored if the goal is to eradicate the malignancy in vivo before proceeding to clinical trials^67^. Thus, towards removing this obstacle, the uptake of GNPs into CAFs in vitro was measured and compared to that of normal cells and cancer cells. There is an observed increase in the uptake of GNPs into CAFs relative to the other cell lines, which translated to an increased dose enhancement into CAFs vs HeLa and FBs as measured with a 53BP1 and ${\gamma H}2{\text{AX}}$ foci immunofluorescent assay after irradiation with a 2 Gy dose. Moving towards a 3D in vitro model such as spheroids could prove beneficial to elucidating the effect of radiation on CAFs further effects^68^. Overall, this study unveils the feasibility of using GNPs as a tool to cross barriers within the TME to yield a better therapeutic outcome in future cancer RT.

## Materials and methods

### Cells and materials

HeLa, fibroblast (Hs. 895.Sk), and cancer associated fibroblasts (Hs 895.T) were purchased from the American Type Culture Collection (ATCC) and the catalogue numbers are CCL-2, CRL-7636, and CRL 7637, respectively. All cell lines were cultured in high glucose Dulbecco’s Modified Eagle Medium (DMEM; Gibco) supplemented with 10% fetal bovine serum (FBS; Gibco), 1% penicillin/streptomycin (Gibco), and 4 mM of GlutaMax (Gibco), and trypsin–EDTA(Gibco) was used for cell dissociation. For confocal experiments, FluoroBrite DMEM (Gibco) was supplemented with 10% FBS and 1% penicillin/streptomycin after staining with CellLight Tubulin-GFP (BacMam 2.0, Thermo-Fisher), while the cells were grown on 3 cm coverslip-bottom dishes supplied by MatTek. Cells were fixed using paraformaldehyde (PFA; Sigma Aldrich). For synthesis of GNPs, chloroauric acid (${\text{HAuCl}}_{4} \cdot 3{\text{H}}_{2} {\text{O}}$; Sigma Aldrich) and sodium citrate tribasic dihydrate (HOC(COONa)(CH_2_COONa)_2_ 2H_2_O; Sigma Aldrich) were used. Both polyethylene glycol (PEG)-thiol and PEG-thiol-Cy5 were purchased from Nanocs. The peptide NH_2_-Cys-Lys-Lys-Lys-Lys-Lys-Lys-Gly-Gly-Arg-Gly-Asp-Met-Phe-Gly-SH was purchased from AnaSpec. For proliferation assays, plates were covered with Breathe-Easy sealing membranes (Sigma-Aldrich) and treated with PrestoBlue (Invitrogen). For immunofluorescence assays, primary antibodies anti-phospho-Histone H2A.X (Ser139; Millipore Sigma) and phospho-53BP1 (Ser1778; Cell Signaling Technology) and secondary antibodies donkey anti-Mouse, Alex Fluor 647 (ThermoFisher) and donkey anti-Rabbit, Alexa Fluor 488 (ThermoFisher) were employed. Bovine serum albumin (BSA; Invitrogen), Triton-X (Sigma-Aldrich), and Tween-20 (Sigma-Aldrich) were also used in the immunofluorescence assay.

### Synthesis, surface modification, and characterization of GNPs

To prepare spherical GNPs of approximately 15 nm in diameter, a citrate reduction method was used^70^. On a hotplate, 300 μL of 1% chloroauric acid (${\text{HauCl}}_{4}$ ) was added to 30 mL of double distilled water and brought to 100 °C while spinning vigorously. Once boiling, 1 mL of 1% sodium citrate tribasic dihydrate $\left( {{\text{HOC}}\left( {{\text{COONa}}} \right)\left( {{\text{CH}}_{2} {\text{COONa}}} \right)_{2} \cdot2{\text{H}}_{2} {\text{O}}} \right)$ was added to the mixture. Upon addition, the solution’s color changes from clear to black to a ruby red, signifying formation of GNPs. The solution was then brought back to room temperature while spinning.

The GNPs were PEGylated using 2 kDa PEG-thiol at a ratio of 1 PEG molecule per nm^2^ of surface area, assuming a perfect sphere. For each 15 nm PEGylated GNP (${\text{GNP}}_{{{\text{PEG}}}}$), 707 PEG molecules were added to the GNP solution and mixed. For confocal imaging, ${\text{ GNP}}_{{{\text{PEG}} - {\text{Cy}}5}} { }$ was synthesized with a mix of the 2 kDa PEG and a 3.2 kDa PEG-thiol-Cy5 in equal proportions, with a total of 707 PEG per GNP. Both complexes were than tagged with a peptide containing integrin binding domain RGD (NH_2_-Cys-Lys-Lys-Lys-Lys-Lys-Lys-Gly-Gly-**Arg-Gly-Asp**-Met-Phe-Gly-SH) at 1 molecule per every 2 PEG molecules. A schematic of the ${\text{GNP}}_{{{\text{PEG}} - {\text{RGD}}}}$ complex can be seen in Fig. 2a.

GNPs, ${\text{GNP}}_{{{\text{PEG}}}}$, and ${\text{GNP}}_{{{\text{PEG}} - {\text{RGD}}}}$ complexes were characterized via ultraviolet–visible (UV–VIS) spectrometry (Perkin Elmer λ 365 Spectrophotometer) for size and concentration estimates, as well as DLS and ζ-potential (Anton Paar LiteSizer 500) for measurement of the hydrodynamic radius and surface charge. Stability of the ${\text{GNP}}_{{{\text{PEG}} - {\text{RGD}}}}$ was measured using DLS in phosphate buffered saline as seen in Supplement S1E.

The ${\text{GNP}}_{{{\text{PEG}} - {\text{RGD}}}}$ complex were imaged using darkfield microscopy and HSI (CytoViva). Darkfield microscopy in combination with HSI allow for spectral information to verify the presence of GNPs. Furthermore, to verify polydispersity and size distribution of the ${\text{GNP}}_{{{\text{PEG}} - {\text{RGD}}}}$ complex, TEM (Hitachi HF-3300 V) images were acquired. The average diameter of the core of the GNPs is calculated using ImageJ software.

### Cellular uptake and retention of ${\text{GNP}}_{{{\text{PEG}} - {\text{RGD}}}}$ complex

Incubation with ${\text{GNP}}_{{{\text{PEG}} - {\text{RGD}}}}$ was completed at a concentration of 0.2 nM for all stated experiments. All three cell lines were plated at a density of $1 \times 10^{4}$ cells per well in a 6-well plate. Once the cells were adhered, they were all incubated with media containing ${\text{GNP}}_{{{\text{PEG}} - {\text{RGD}}}}$ at 37 °C with 5% ${\text{O}}_{2}$. After 24 h of incubation, the cells were then rinsed with phosphate buffered saline (PBS) three times and trypsinized using 0.25% trypsin–EDTA for 5 min. The cells were counted using a Coulter Counter (Z2 Coulter; Beckman Coulter) for GNP quantification per cell. The average volume per cell was given from a distribution of the population of cells measured using the Z2 Coulter Counter. For the retention study, at 24 h after incubation with ${\text{GNP}}_{{{\text{PEG}} - {\text{RGD}}}}$, the media was replaced and incubated for 24 h.

To measure the gold content for each condition, the cells were treated with 65% aqua regia (3:1 ratio of ${\text{HCl:HNO}}_{3} \,({\text{VWR}}))\,{\text{in}}\,{\text{a}}\,200\,{ }^{{\text{o}}} {\text{C}}$ mineral oil bath for a minimum 1 h. Small amounts of hydrogen peroxide were added afterwards to ensure complete digestion of the cells and GNPs. These samples were then diluted down to 2.5% v/v acid content in deionized water and the gold content was quantified using inductively coupled plasma mass spectrometry (ICP-MS; Agilent 8800 Triple Quadrupole).

Calculation of the GNP concentration from the absolute number of Au atoms was done using the following equations:

$$\begin{aligned} \frac{{{\text{Number}}\,{\text{of}}\,{\text{Au}}\,{\text{atoms}}}}{{{\text{GNP}}}}\left[ {\text{U}} \right] & = \frac{{{\text{Number}}\,{\text{of}}\,{\text{atoms}}\,{\text{per}}\,{\text{unit}}\,{\text{cell}} \cdot {\text{Volume}}\,{\text{of}}\,{\text{GNP}}}}{{{\text{Volume}}\,{\text{of}}\,{\text{unit}}\,{\text{cell}}}} \\ & = 4 \cdot \frac{{\frac{4}{3}{\uppi }\left( {\frac{{\text{D}}}{2}} \right)^{3} }}{{{\text{a}}^{3} }} \\ & = \frac{2}{3}{\uppi }\left( {\frac{{\text{D}}}{{\text{a}}}} \right)^{{3{ }}} \\ \end{aligned}$$

where D = core diameter of the GNP, a = length of a unit cell, 0.408 nm. GNPs that are synthesized using the citrate reduction method assemble into a face-centered cubic structure, which as a lattice containing 4 atoms per unit cell^71^. This assumes a homogenous size of GNPs, which is verified through TEM imaging.

Using this, the number of GNPs per sample is calculated from:

$$= {\text{conc}}.\,{\text{measured }}\left[ {\frac{{\text{g}}}{{\text{L}}}} \right] \cdot {\text{volume }}\left[ {\text{L}} \right] \cdot \frac{1}{{{\text{molar}}\,{\text{weight}}\,{\text{of}}\,{\text{Au }}}}\left[ {\frac{{{\text{mol}}}}{{\text{g}}}} \right] \cdot {\text{N}}_{{\text{A}}} \left[ {\frac{{\text{Au }}}{{{\text{mol}}}}} \right] \cdot \frac{1}{{\text{U}}}\left[ {\frac{{{\text{GNP}}}}{{\text{Au }}}} \right]$$

where ${\text{N}}_{{\text{A}}}$ is Avogadro numbers. The number of GNPs per cell is then calculated by dividing the number of GNPs by the number of cells, assuming a homogenous distribution of GNPs in the cell population.

### Preparation of cells for darkfield and confocal imaging

To prepare cells for darkfield imaging, all cell lines were plated in a six-well plate with glass coverslips placed on the bottom of each well. The cells were then treated as described previously using ${\text{ GNP}}_{{{\text{PEG}} - {\text{RGD}}}}$ with a 24-h uptake time point and a 24-h retention time point. Upon completion of each experiment, the cells were rinsed three times with PBS and fixed using 4% PFA for 20 min at 37 °C. The cover slips were then removed from each well and mounted to a glass slide using Prolong Glass Antifade Mountant. Each sample was imaged using darkfield microscopy and HSI (CytoViva) under a 60X objective.

Live-cell imaging of the distribution and uptake of the ${\text{GNP}}_{{{\text{PEG}} - {\text{RGD}}}}$ complex was performed using confocal microscopy (Zeiss LSM 980) using a 60X oil immersion lens. ${\text{GNP}}_{{{\text{PEG}} - {\text{RGD}}}}$ complexes had PEG-Cy5 (excitation 633 nm, emission filter 650 nm LP) conjugated as previously mentioned. To see general structure of the cell, microtubules were stained with a viral transfection stain (CellLight Tubulin-GFP), which contains DNA coding for an α-tubulin/GFP construct.

To prepare cells for live-cell confocal imaging, cells were plated on 3 cm coverslip-bottom dishes in FluoroBrite media. The cells were incubated in the viral stain for > 24 h prior to treatment with fluorescent ${\text{GNP}}_{{{\text{PEG}} - {\text{RGD}}}} .$ After incubation with GNPs, the cells were imaged after 24 h of uptake and after 24 h of retention. All conditions including acquisition settings used between experiments was maintained constant.

### Radiation treatment

Cells were plated and incubated with GNPs 24 h prior to any radiation treatment. The plates were then placed between two 30 cm × 30 cm × 5 cm solid water blocks at isocenter of a 6 MV medical linear accelerator (Varian TrueBeam) and given a 2 Gy photon dose from a single 28 cm × 28 cm field. To ensure consistency, control cells were brought to the linear accelerator but not irradiated.

### Doubling time assay

Cells were plated at a density of $2.5 \times 10^{3}$ to $10 \times 10^{3}$ cells/well in black-walled 96-well plates and covered with a breathable membrane to reduce evaporation effects. The cells were then irradiated as described above. An hour after irradiation, a row of cells had the membrane removed and rinsed with PBS, followed by incubation in 10% PrestoBlue in media, v/v, for 1 h. PrestoBlue is a resazurin-based dye which measures viable, metabolically active cells via the reduction of resazurin to resorufin and can be detected fluorometrically^72^. Subsequent measurements were taken every day for the first three days, followed by measurements every second day until confluency was reached.

### Immunofluorescence assay

Cells were grown on glass coverslips in six-well plates for 24 h prior to experimentation. After 24 h of endocytosis of ${\text{GNP}}_{{{\text{PEG}} - {\text{RGD}}}}$ complexes, the cells were treated with radiation as described previously, and incubated for 24 h at 37 °C. The cells were fixed with 4% PFA for 5 min at room temperature followed by two PBS washes for 5 min each. The cells were then blocked to reduce background noise using 2% BSA/0.1% Triton-X in PBS for 20 min. The two primary antibodies ${\gamma H}2{\text{AX }}$ and 53BP1 were diluted 1:200 in 0.5% BSA/0.1% Triton-X/PBS, while the secondary antibody was diluted 1:500 in 0.5% BSA/0.1% Triton-X/PBS. The coverslips were placed cells-down into 50 µL of a combination of the two primary antibodies on parafilm and incubated for an hour, followed by washing with PBS for five minutes. The cells were then rinsed twice with 0.5% BSA/0.175% Tween-20/PBS for 5 min. On a new parafilm, the cover slips were placed cells-down in 50 µL of both secondary antibodies and incubated in the dark for 30 min. Finally, the cells were rinsed in PBS, dried, mounted to glass coverslips with Prolong Glass, and imaged with confocal microscopy as described previously.

### Statistical analysis

A t-test correcting for multiple comparisons using the Holm-Sidak method was preformed using GraphPad Prism 8. A *p *value < 0.05 was considered statistically significant. Experiments were repeated three times and the data presented is the average, for all experiments.

## Supplementary information

Supplementary information
