# Supplementary material for: Gold nanoparticle mediated radiation response among key cell components of the tumour microenvironment for the advancement of cancer nanotechnology
Source: Sci Rep. 2020 Jul 21;10:12096. doi: 10.1038/s41598-020-68994-0 (PMC7374632; doi:10.1038/s41598-020-68994-0)
Supplement: Supplementary file 1 — Supplementary information [file 41598_2020_68994_MOESM1_ESM.pdf]

Gold nanoparticle mediated radiation response among key cell components of the tumour microenvironment for the advancement of cancer nanotechnology

Kyle Bromma[1], *Leah Cicon*[1], *Wayne Beckham*[1,2], *and Devika B. Chithrani*[1,2,3, 4*]

[1] Department of Physics and Astronomy, University of Victoria, Victoria, BC, Canada.

[2] British Columbia Cancer Agency, Victoria, BC, Canada.

[3] Centre for Advanced Materials and Related Technologies (CAMTEC), University of Victoria, Victoria, BC, Canada.

[4] Centre for Biomedical Research, University of Victoria, Victoria, BC, Canada.

Supplementary section S1: Characterization of GNPs

A

Functionalization	Peak Absorption Wavelength (nm)	Hydrodynamic Diameter (nm)	Zeta Potential (mV)
GNP	520.05 ± 0.05	18.02 ± 0.36	-42.61 ± 1.98
GNP+PEG	520.95 ± 0.05	$21.34 \pm .43$	-25.19 ± 1.62
GNP+PEG+RGD	523.07 ± 0.05	29.3 ± 0.59	-0.8 ± 0.39

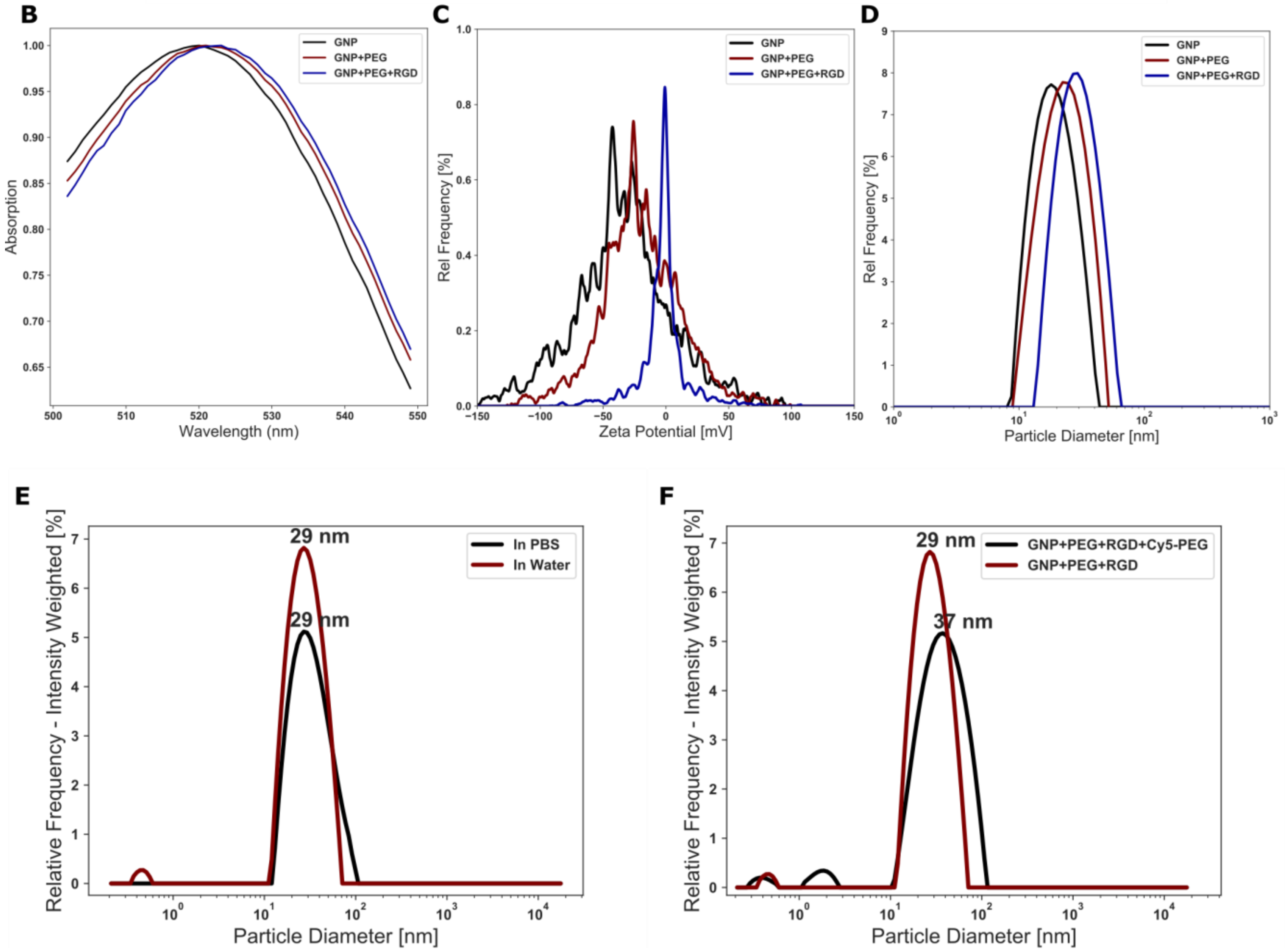

Supplementary Figure S1. A) Characterization data for GNPs, $\mathbf{GNP_{PEG}}$, and $\mathbf{GNP_{PEG+RGD}}$ from UV-Vis, DLS, and zeta potential. All sample measurements in (B) UV-Vis, (C) DLS, and (D) zeta potential are plotted. (E) DLS measurements of $\mathbf{GNP_{PEG+RGD}}$ in phosphate buffered saline (PBS) as compared to the base solvent of water. (G) DLS measurements of fluorescent $\mathbf{GNP_{PEG+RGD}}$ tagged with PEG-thiol-Cy5 as compared to normal $\mathbf{GNP_{PEG+RGD}}$.

Supplementary section S2: Uptake and retention of GNPs

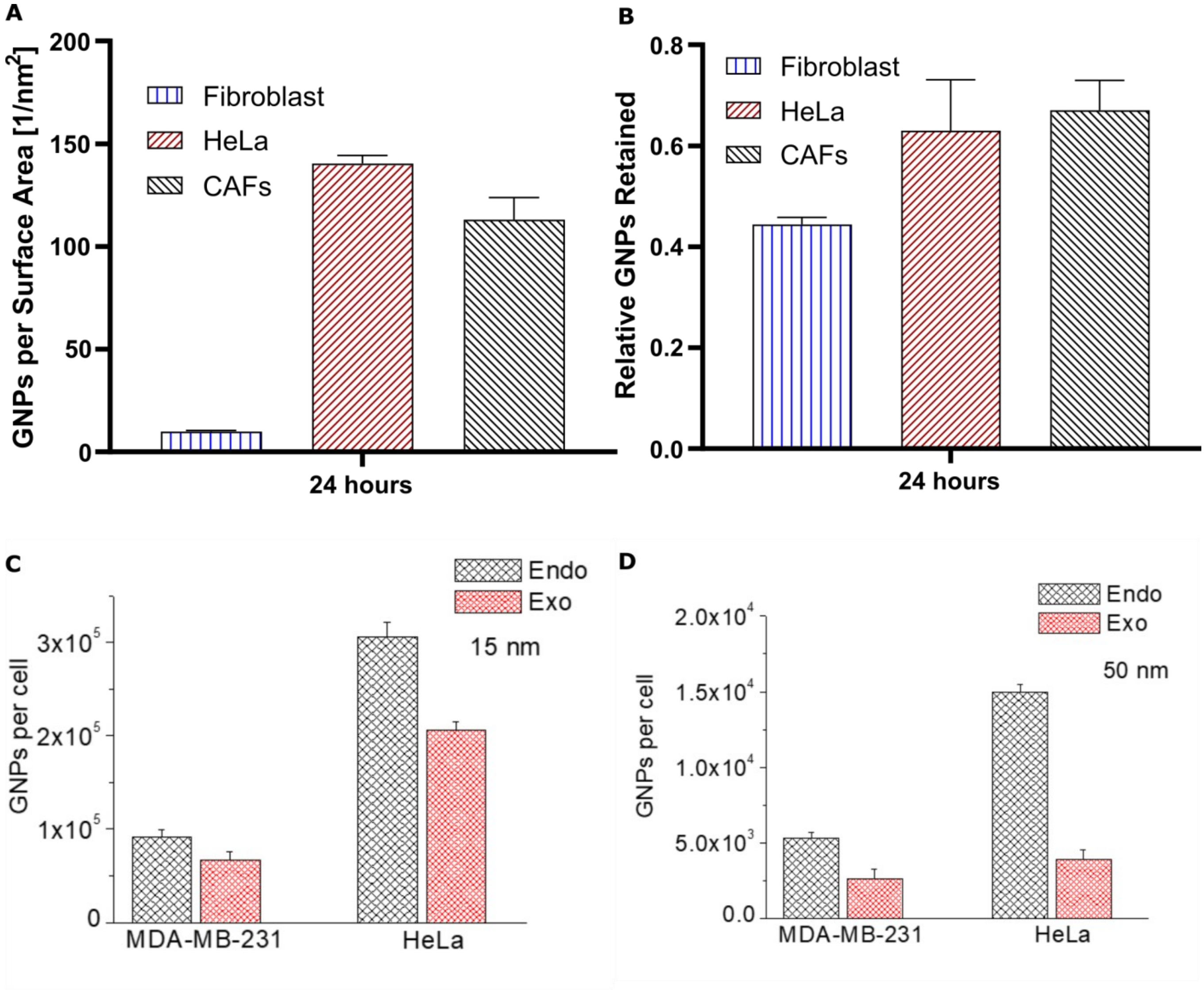

Supplementary Figure S2. (A) Surface area normalized uptake of GNPs. (B) Relative retention of GNPs after 24 hours. Uptake of (C) smaller 15 nm GNPs and (D) larger 50 nm GNPs in two cancer cell lines, HeLa and MDA-MB-231, a breast cancer cell line, after 4 hours of uptake (endo) and after another 24 hours of release (exo).

Supplementary Section S3: Microtubule transport of nanoparticles

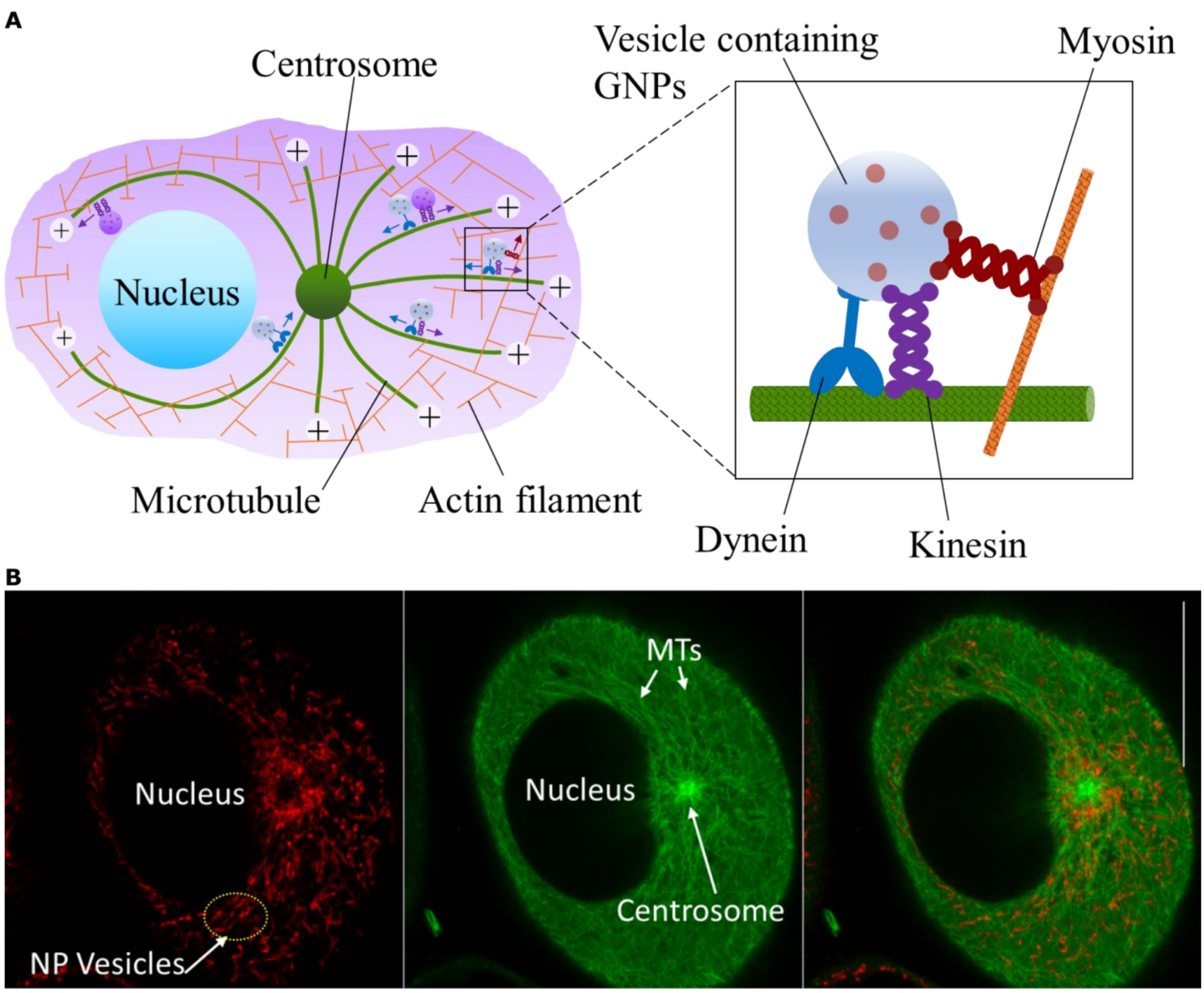

Supplementary Figure S3. (A) Transport of vesicles containing the GNPs along the cellular microtubule (MT) network. MTs are long tubulin polymers, often anchored at the centrosome, where transport of vesicles is controlled via motor proteins, dynein and kinesin (inset figure). (B) Live cell imaging of vesicles containing GNPs (marked in red; left), MTs (marked in green; center), and the two images merged(right). Scale bar is 20 μm.

Supplementary section S4: Proliferation of cells after radiation

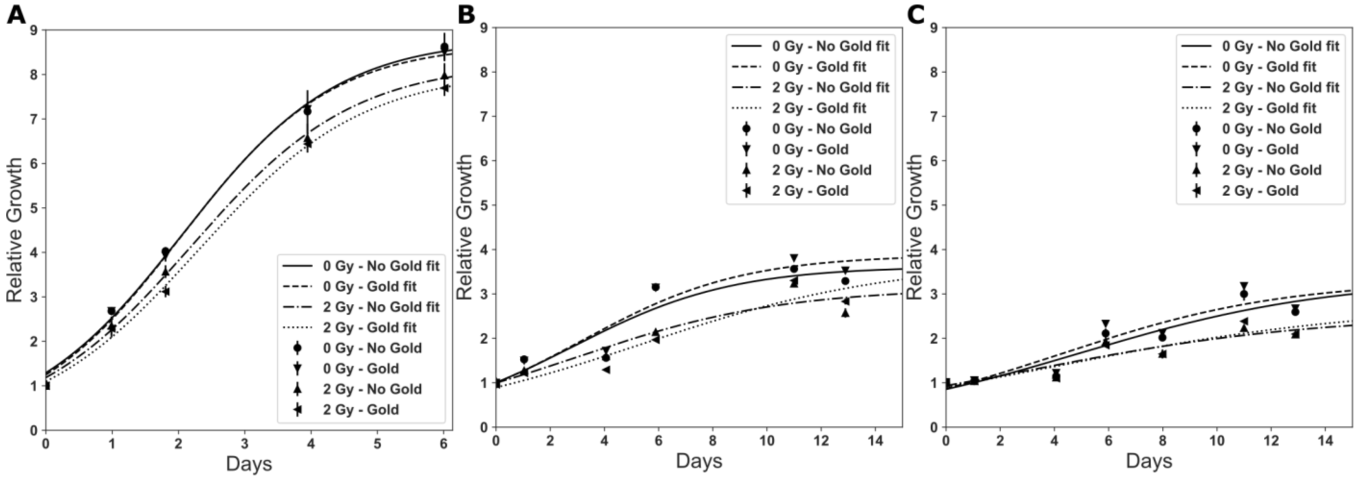

D

Sample	HeLa			Fibroblast			CAFs		
	Param 'A'	Param 'λ'	Param 'μ'	Param 'A'	Param 'λ'	Param 'μ'	Param 'A'	Param 'λ'	Param 'μ'
0 Gy - No Gold	8.82 ±0.40	-0.28 ±0.21	1.88 ±0.19	3.62 ±0.57	-3.06 ±2.59	0.31 ±0.13	3.34 ±1.13	-4.31 ±2.59	0.18 ±0.05
0 Gy - Gold	8.69 ±0.33	-0.24 ±0.18	1.91 ±0.17	3.88 ±0.50	-2.79 ±1.98	0.33 ±0.11	3.31 ±0.93	-4.00 ±2.80	0.20 ±0.07
2 Gy - No Gold	8.27 ±0.27	-0.26 ±0.14	1.69 ±0.11	3.13 ±0.63	-4.73 ±3.12	0.20 ±0.08	2.54 ±0.89	-7.90 ±4.60	0.12 ±0.05
2 Gy - Gold	8.10 ±0.30	-0.18 ±0.15	1.63 ±0.11	3.74 ±1.44	-3.90 ±2.44	0.20 ±0.062	2.76 ±1.23	-7.49 ±4.04	0.12 ±0.04

E

	**HeLa - T_d [hrs]**	**Fibroblast - T_d [hrs]**	**CAF - T_d [hrs]**
**0 Gy - No Gold**	19.40	49.70	77.00
**0 Gy - Gold**	19.38	49.29	76.5
**2 Gy - No Gold**	20.38	63.64	90.25
**2 Gy - Gold**	20.63	71.2	96.3

Supplementary Figure S4. (A-C) Relative growth patterns for (A) HeLa, (B) NFs, and (C) CAFs. The data is fit with a biologically reparametrized logistic function, where the parameters can be found in (D). Calculated doubling times from the parameters can be found in (E).

Supplementary section S5: Immunofluorescent DSB assays

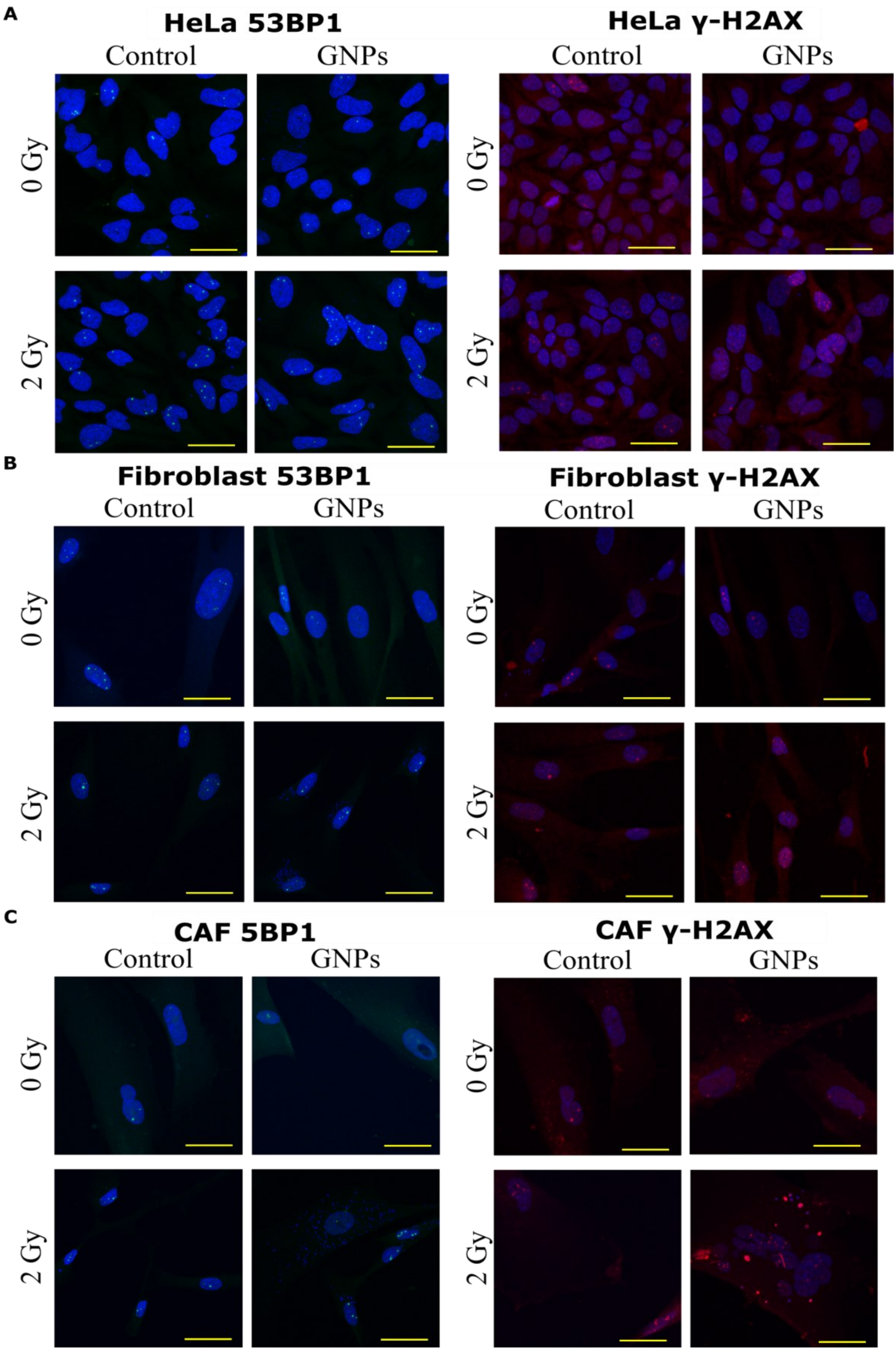

Supplementary Figure S5. Example images of control and irradiated (A) HeLa cells, (B) NFs, and (C) CAFs treated with and without GNPs, then stained for 53BP1 and $\boldsymbol{\gamma}-\mathbf{H2AX}$ foci. The nuclei are marked in blue while 53BP1 foci are marked in green and $\boldsymbol{\gamma}-\mathbf{H2AX}$ foci are marked in red. Scale bar = 20 µm.

Supplementary section S6: Immunofluorescent assay and confirmation of encapsulated GNPs

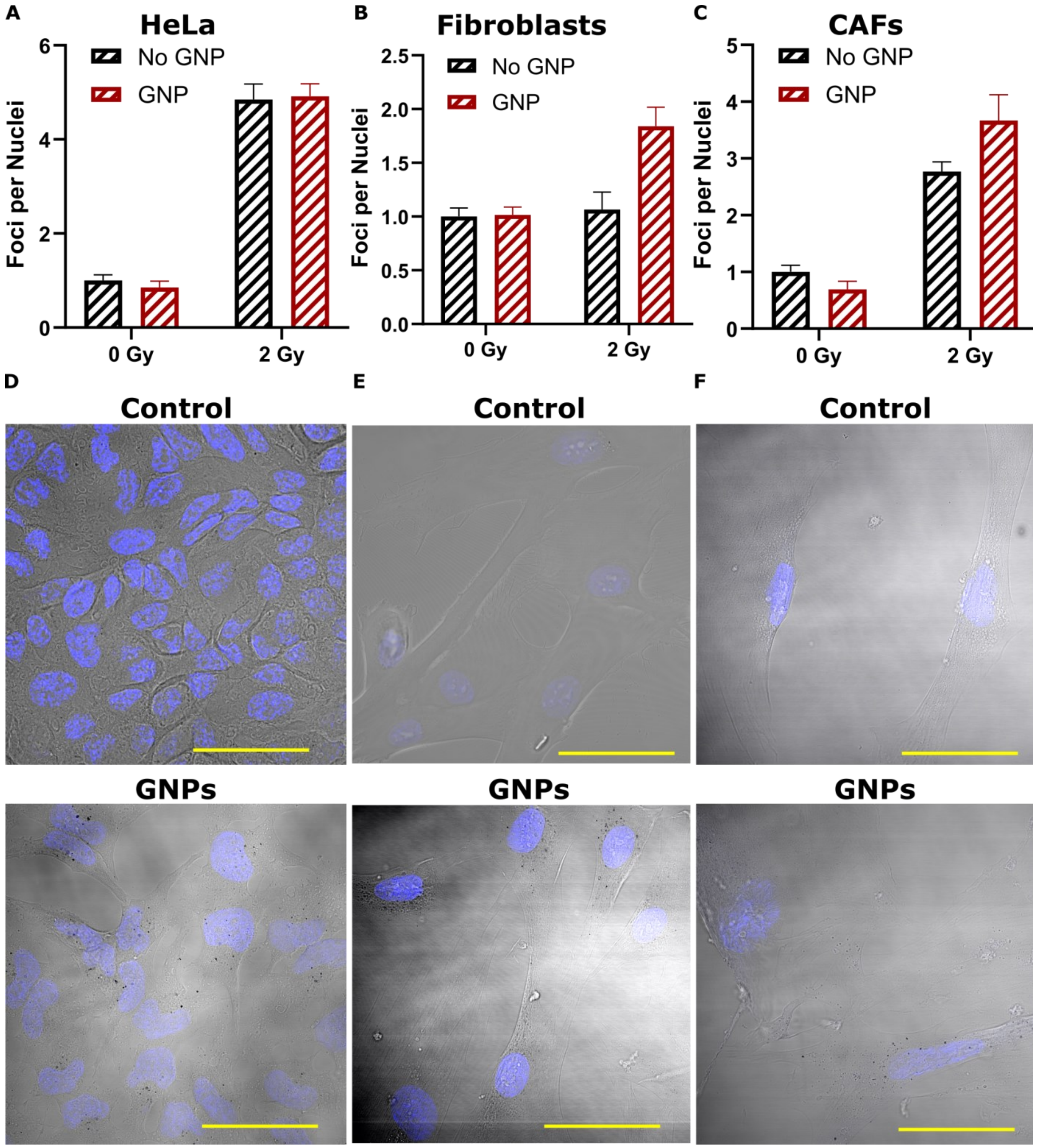

Supplementary Figure S6. (A-C) Quantification of $\gamma - \mathbf{H2AX}$ Foci from a minimum of 50 different imaged nuclei for (A) HeLa, (B) NFs, and (C) CAFs. (D-F) are pseudo brightfield images of cells with and without gold for (D) HeLa, (E) NFs, and (F) CAFs. In the bottom images, the black dots present are clusters of GNPs aggregated in the cells. Scale bar = 20 µm.

Supplementary section S7: Experimental clonogenic assay results for HeLa

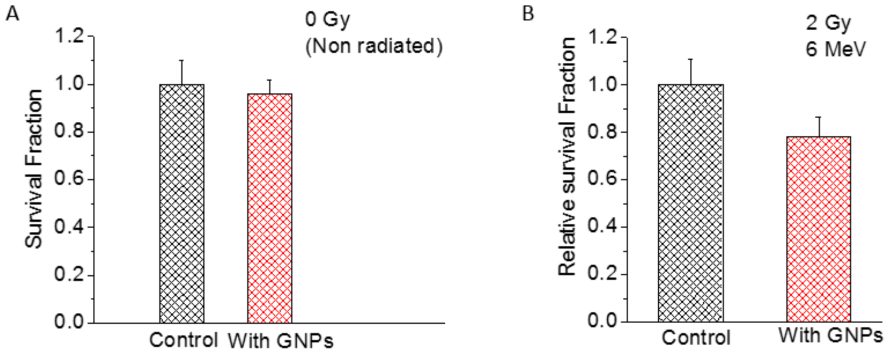

Supplementary Figure S7. Experimental results of clonogenic assay for HeLa. (A-B) Comparison of survival fractions for non-radiated and radiated cells, respectively.

Supplement section S8: Summary of gold nanoparticles as a model nanotechnology

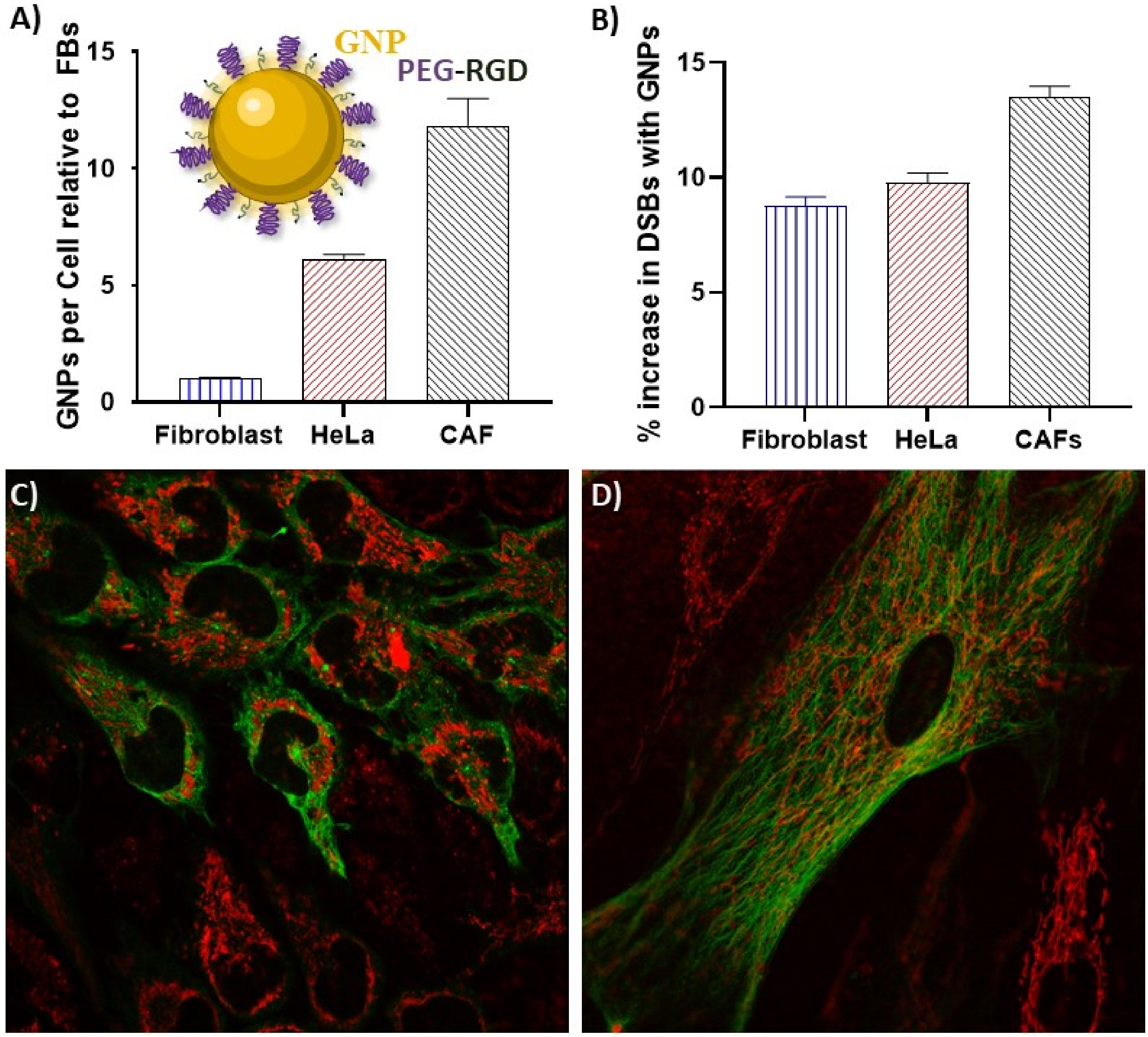

Supplementary Figure S8. Gold nanoparticles as a model nanotechnology in the treatment of cancer. (A) Uptake of gold nanoparticles (GNPs; inset) into cancer associated cells such as HeLa and cancer associated fibroblasts (CAFs) are far larger than that observed into normal fibroblasts. This translates to (B) an increase of double strand breaks (DSBs) following radiation. (C) HeLa and (D) CAF cells imaged with confocal imaging. The microtubule structure is labelled in green while the GNPs are labelled in red.
